# Ribosome hibernation in zinc-starved *Mycobacterium abscessus* confers amikacin tolerance

**DOI:** 10.1128/spectrum.00378-26

**Published:** 2026-07-06

**Authors:** Ryan Z. Treen, Austin J. Fox, Yunlong Li, Kelley Hurst-Hess, Nilesh K. Banavali, Pallavi Ghosh, Rajendra K. Agrawal, Anil K. Ojha

**Affiliations:** 1 Division of Genetics, Wadsworth Center, NY State Department of Health 1094, Albany, New York, USA; 2 Department of Biomedical Sciences, University at Albany 1084, Albany, New York, USA; 3 The RNA Institute, University at Albany 1084, Albany, New York, USA; Institut de pharmacologie et biologie structurale, Toulouse, France

**Keywords:** *Mycobacterium abscessus*, ribosome remodeling, antibiotic tolerance, NTM

## Abstract

Intrinsic and acquired antibiotic resistance in *Mycobacterium abscessus* present unique challenges in treatment of its infections, which are rapidly emerging as a significant public health threat. The majority of clinically relevant antibiotics used against *M. abscessus* infections target the ribosome, which undergoes remodeling and hibernation in *Mycobacterium smegmatis* and *Mycobacterium tuberculosis* in response to zinc-limiting conditions. Ribosome remodeling involves replacement of multiple zinc-binding C+ ribosomal proteins with a CXXC motif by their respective C− paralogs lacking the motif, whereas ribosome hibernation involves recruitment of mycobacterial protein Y (Mpy) to the mRNA decoding center on the 30S subunit. Here, we report that zinc-responsive ribosome remodeling and hibernation are conserved in *M. abscessus*. We further demonstrate that Mpy binding suppresses translation and preserves ribosome abundance under zinc-limited conditions, while conferring tolerance to the aminoglycoside amikacin. Systematic biochemical analyses demonstrate that amino acid residues of Mpy that are critical for its interaction with the ribosome are also essential for Mpy stability in the cytosol. Together, these findings demonstrate amikacin tolerance as an important outcome of ribosome hibernation in *M. abscessus*.

**IMPORTANCE:**

*Mycobacterium abscessus* causes life-threatening infections in people with underlying health conditions. The treatment regimens for *M. abscessus* infections are months-long and include several ribosome-targeting antibiotics, such as amikacin. The long regimens are primarily attributed to intrinsic drug resistance in the pathogen. However, mechanisms of resistance for several of the antibiotics remain unclear. Here, we show that ribosome hibernation in *M. abscessus* by Mpy under zinc-starved conditions, which likely prevail in hosts, is a key determinant of amikacin tolerance. Thus, Mpy is a potential target for potentiating amikacin activity against *M. abscessus*.

## INTRODUCTION

*Mycobacterium abscessus* is a rapidly growing, nontuberculous mycobacterial (NTM) species ubiquitously prevalent in the environment and can cause soft-tissue or skin infections in individuals with underlying health conditions (1–3). Infections with *M. abscessus* constitute an emerging public health threat, particularly in patients with cystic fibrosis or other chronic obstructive pulmonary disease conditions (4–7). Comparison of lung function defects resulting from various common bacterial infections in cystic fibrosis patients reveals that *M. abscessus* caused the most severe defects, surpassing *M. avium* and *P. aeruginosa* (8).

Treatment of *M. abscessus* infections typically consists of an oral macrolide such as clarithromycin or azithromycin, combined with a parenteral agent such as amikacin, imipenem, or cefoxitin (9). Many of these frontline antibiotics exert their antibacterial effects by binding to the mycobacterial ribosome, which undergoes significant changes in *Mycobacterium tuberculosis* and *Mycobacterium smegmatis* in response to zinc limitation—a condition likely encountered in host lungs. Evidence of zinc-starvation responses in *M. tuberculosis* in animal models as well as in sputa of TB patients underscores the clinical significance of zinc-responsive changes in the mycobacterial ribosomes (10–13).

Specifically, the ribosome is remodeled under moderately limiting, yet growth-permissive concentrations of free zinc, in which each ribosomal (r-) protein with the zinc-binding CXXC motif, therefore called C+ r-protein, is replaced by its C− paralog without the CXXC motif (10, 13–17). Under growth-restrictive concentrations of free zinc, the remodeled ribosome is targeted for hibernation by mycobacterial-specific protein Y (Mpy) (10). Ribosome remodeling occurs at a higher concentration of free zinc than that required for recruitment of Mpy to the ribosome (10). At free zinc concentrations that permit ribosome remodeling but are not low enough for ribosome hibernation, the Clp protease system degrades the Mpy recruitment factor (Mrf), a protein that promotes Mpy recruitment to the ribosome (10). Since Mrf is degraded during ribosome remodeling, ribosome remodeling is uncoupled from hibernation through distinct levels of severity in zinc starvation (10). However, zinc-responsive ribosome remodeling and ribosome hibernation have not yet been elucidated in *M. abscessus*, and we reasoned that an investigation would provide insights for effective therapeutic strategies.

Here, we investigate ribosome hibernation in *M. abscessus* and describe a systematic approach that addresses the extent of Mpy recruitment to *M. abscessus* ribosomes under high- and low-zinc ribosomes from various growth phases. We finally report the role of Mpy-dependent ribosome hibernation in tolerance of *M. abscessus* to the frontline drug amikacin used to treat its infection.

## RESULTS

### Zinc-responsive ribosome remodeling in *M. abscessus*

**Fig 1:**
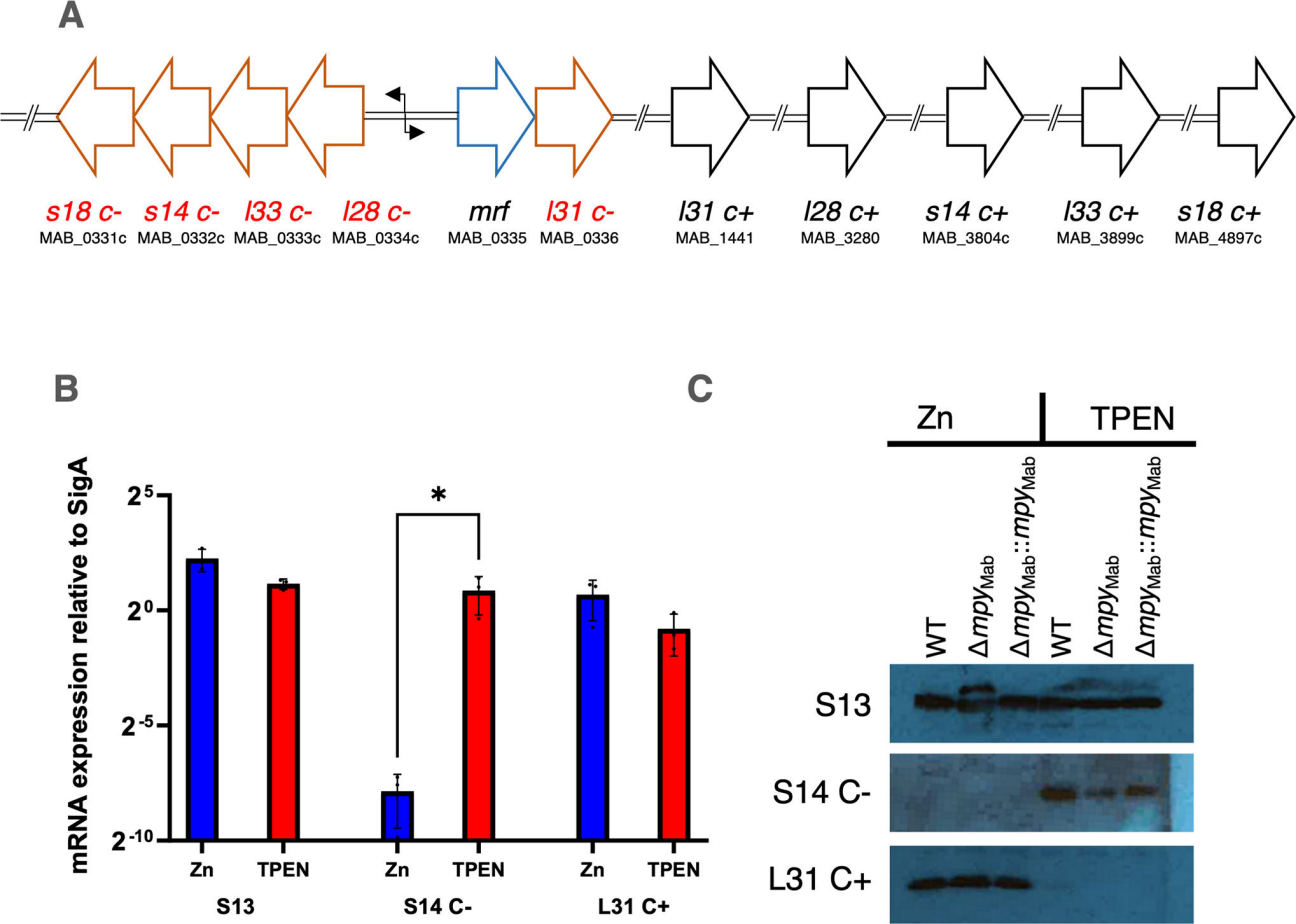
Ribosome remodeling in *M. abscessus*. (**A**) Genomic organization of C+ and C− ribosomes in *M. abscessus*. Genes encoding *c*− r-proteins are depicted in red, while their C+ counterparts in black. The gene for Mpy recruitment factor (*mrf*) included within c− operon, is shown in blue. The arrows indicate the Zur-regulated bidirectional promoters of the operon, which is repressed under high-zinc conditions. (**B**) Zinc-responsive transcriptional de-repression of *c*− operon in *M. abscessus* from its native promoter, measured by the abundance of *S13*, *S14_C−_*, and *L31*_C+_ mRNAs relative to SigA levels in cells grown in high- (1mM ZnSO_4_) and low-zinc (2 μM TPEN) Sauton’s medium. Data represent mean ± SD from three independent experiments. *P < 0.05 (unpaired two-tailed Student’s *t*-test). (**C**) Levels of S14_C−_ and L31_C+_ in 70S ribosomes purified from *M. abscessus* wild-type (WT) and its isogenic Δ*mpy* or complemented (Δ*mpy*_Mab_::*mpy*_Mab_) strains cultured in low- or high-zinc Sauton’s medium for 48 h; 2.4 pmol of pure ribosomes resolved on a 15% SDS-PAGE gel was transferred onto a PVDF membrane, which was then probed with anti-S14_C−_ (Mab_0332c), anti-L31_C+_ (Mab_1441) antibodies. S13 (Mab_3773c) was probed as a loading control.

Like *M. smegmatis*, an operon of paralogous pairs of five r-proteins (L28, L31, L33, S14, and S18) that differ by the presence or absence of CXXC motif are encoded by *M. abscessus* (Fig. 1A). We sought to investigate if and how the expression of these r-proteins in *M. abscessus* responds to zinc. Total RNA from 2-day-old high- and low-zinc cultures of *M. abscessus* was extracted*,* and levels of mRNA corresponding to a representative C+ and C− gene*, l31_C+_* and *s14_C-_* respectively, along with an invariant *s13* gene*,* were quantified using RT-PCR. While the expression of *l31_C+_* was constitutive in high- and low-zinc conditions, expression of *s14_C−_* in the low-zinc culture was ~500-fold greater when compared with the high-zinc cultures (Fig. 1B). The change in the S14_C−_ mRNA level was further consistent with the change in the corresponding protein level in the 70S ribosomes purified from high- and low-zinc cultures of *M. abscessus* (Fig. 1C). Interestingly, although L31_C+_ mRNA was relatively unchanged in high- and low-zinc conditions (Fig. 1B), L31_C+_ protein was undetectable in the low-zinc culture. The unchanged expression of L31_C+_, and likely the other C+ protein paralogs, in high- and low-zinc cultures is consistent with our previous findings in *M. smegmatis* (13), suggesting that its absence in the ribosome under low-zinc condition is perhaps because it is outcompeted by the C− paralog during ribosome assembly. Together, these *in vitro* data indicate that *M. abscessus* constitutively expresses C+ r-proteins, while transcriptionally regulating the expression of C− r-proteins in a zinc-responsive manner, likely through a Zur-dependent mechanism as observed in *M. smegmatis* and *M. tuberculosis* (10, 13).

### Zinc-responsive Mpy binding to the ribosome in *M. abscessus*

**Fig 2:**
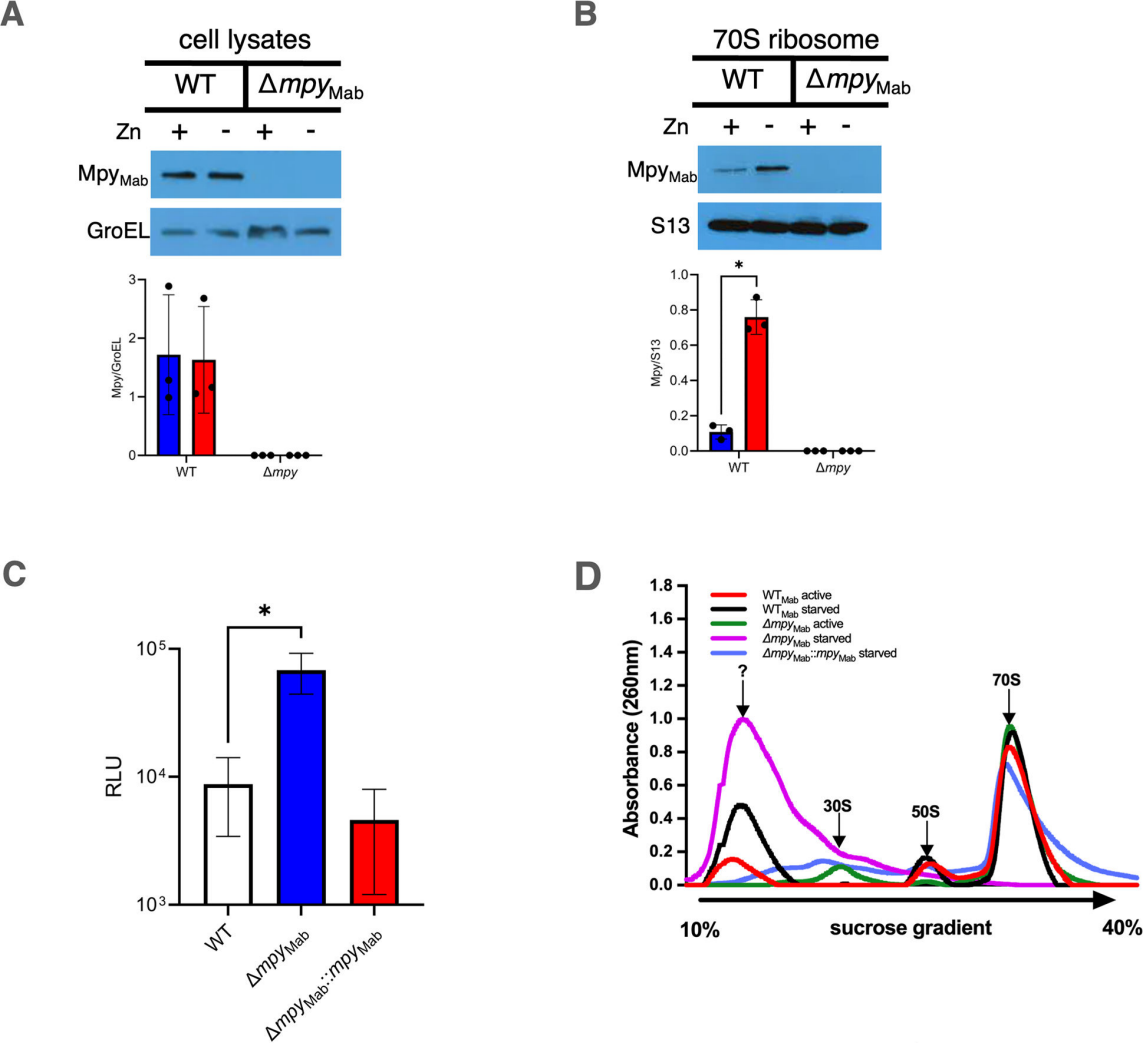
Zinc-responsive ribosome hibernation by Mpy_Mab_ in *M. abscessus*. (**A–B**) Mpy_Mab_ abundance in the cell lysates (**A**) and 70S ribosomes (**B**) from WT and isogenic Δ*mpy*_Mab_ grown in high- and low-zinc Sauton’s medium for 7 days. Mpy_Mab_ was probed with polyclonal antibody raised against *M. smegmatis* Mpy. GroEL and S13 were probed as loading controls for lysates and ribosomes, respectively. Data represent mean ± SD of three biologically independent preparations, * in panel **B** denotes *P* < 0.05 (unpaired two-tailed Student’s *t*-test). (**C**) Nano-luciferase based translation activity of the 70S ribosome purified from wild-type (WT), Δ*mpy*_Mab_, and complemented (Δ*mpy*_Mab_::*mpy*_Mab_) strains cultured in low-zinc Sauton’s medium for 7 days. The activity is reported in relative luminescence unit (RLU). Data represent mean ± SD of three biologically independent preparations of ribosomes. *P < 0.05 (unpaired two-tailed Student’s *t*-test). (**D**) SDG profile of the ribosomes purified from starved WT, Δ*mpy*_Mab_, or Δ*mpy*_Mab_::*mpy*_Mab_ cells. After 7 days of growth in low-zinc Sauton’s medium, cells were starved for 7 days in PBS + 0.05% Tween-80 before purification and analysis of 70S ribosomes. Ribosomes purified from a 7-day actively growing culture of WT and Δ*mpy*_Mab_, were used as references.

MAB_3583c in *M. abscessus*, hereafter referred to as Mpy_Mab_, has 74% protein sequence identity to MSMEG_1878 (Mpy_Msm_). Therefore, using anti-Mpy_Msm_ antibody, we probed the expression of Mpy_Mab_ in *M. abscessus* ATCC19977 and an isogenic Δ*mpy*_Mab_ as control. Consistent with the constitutive expression of Mpy in *M. smegmatis* and *M. tuberculosis*, we observed Mpy_Mab_ signal in the cell lysate of both high- and low-zinc cultures of *M. abscessus* (Fig. 2A), although Mpy_Mab_ signal in the C− ribosome was significantly higher than in the C+ ribosome (Fig. 2B). Moreover, the ribosomes from low-zinc Δ*mpy*_Mab_ culture exhibited significantly higher translation activity *in vitro* compared with the parent wild-type and its isogenic complemented strain of *M. abscessus*, consistent with our previous biochemical and structure-based findings that Mpy binding indeed renders the ribosome functionally inactive (10, 13) (Fig. 2C). Ribosome hibernation has been implicated in maintaining a stable pool of non-translating ribosomes in slow-growing cells (13, 18, 19). We therefore investigated the effect of Mpy_Mab_ binding on the abundance of 70S ribosomes in nutrient-starved *M. abscessus* cells. Analysis of the relative abundance of ribosomes in *M. abscessus* cells, maintained in zinc-chelated PBS for 7 days, revealed a lower abundance of 70S pool in Δ*mpy*_Mab_ compared with the complemented strain (Fig. 2D). Thus, exposure of *M. abscessus* to zinc-chelated PBS induces similar changes in its ribosomes as previously observed in *M. smegmatis* and *M. tuberculosis* (10).

### Growth phase-dependent Mpy-ribosome interaction

**Fig 3:**
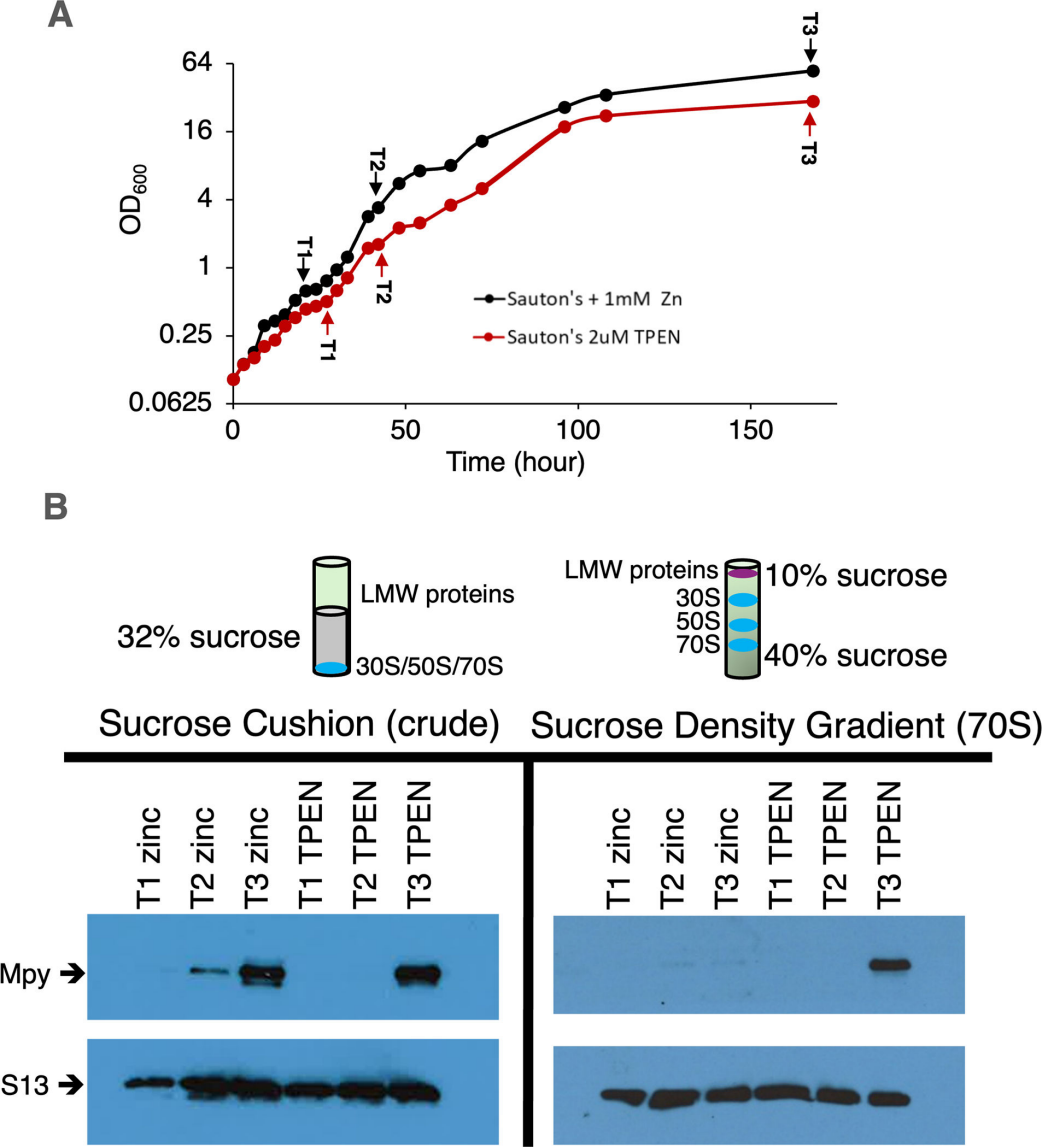
Analysis of Mpy interaction with ribosomes at various growth stages of *M. abscessus* in high- and low-zinc Sauton’s medium. (**A**) Growth curves of wild-type *M. abscessus* in high- or low-zinc Sauton’s medium. Time points at which ribosomes were purified are indicated by arrows. T1 corresponds to the timepoint at which each culture reached an OD_600_ of 0.5, representing logarithmic phase. T2 is taken at 42 h from both high- and low-zinc cultures to represent early stationary phase, and T3 was taken at 7 days for both high and low zinc to represent the late stationary phase. (**B**) Levels of Mpy in the ribosomes purified from high- and low-zinc cultures at T1–T3 stages. Ribosomes were purified either through a 32% sucrose cushion, which sediments 30S, 50S, and 70S particles as well as other macromolecular complexes at the bottom, or 10%–40% SDG that sediments individual components at a distinct layer as shown above the blots.

To gain further insight into when and how Mpy binds to the ribosome in *M. abscessus*, we performed a systematic analysis of Mpy levels in the ribosomes purified from high- and low-zinc cultures at logarithmic, early-stationary, and late-stationary phases. Wild-type *M. abscessus* was grown in high- or low-zinc Sauton’s media for ~24 h (to an OD_600_ of 0.5), 42 h, and 7 days post-inoculation to represent logarithmic, early-, and late-stationary phases, respectively (Fig. 3A). To capture the weaker Mpy-ribosome interactions, we purified ribosomes without a high-concentration ammonium chloride wash, which is typically introduced to remove loosely bound ligands. Crude ribosomes purified from the cultures were centrifuged either through a 32% sucrose cushion to collect the total ribosomes or were fractionated on 10%–40% sucrose density gradient (SDG) to resolve 30S, 50S, and 70S ribosomes (Fig. 3B). Notably, ultracentrifugation through a uniform 32% sucrose solution offers gentler downward force compared with a significant portion of the path length in a 10%–40% SDG. While the sucrose cushion is expected to allow sedimentation of all ribosomal particles and other macromolecular complexes (referred to as total ribosome preparation) at the bottom, 10%–40% SDG sediments 30S, 50S, and 70S ribosomes at distinct equilibrium positions (Fig. 3B). Ribosomes from the logarithmic and early-stationary phases purified using either 32% sucrose cushion or 10%–40% SDG had undetectable to very low levels of Mpy in both high- and low-zinc ribosomes (Fig. 3B). The low Mpy signal during these growth phases can likely be attributed to its inaccessibility to the ribosome particles, most of which are occupied by the canonical protein synthesis ligands, which likely outcompete Mpy for the binding sites on the ribosome (13, 20–24). However, additional regulation of Mpy in the logarithmic and early stationary phases cannot be ruled out. Mpy levels in the total ribosomes from the late stationary phase purified through a 32% sucrose cushion were comparable in both high- and low-zinc cultures (Fig. 3B). However, the 70S ribosome fractionated on 10%–40% SDG had different levels of Mpy in high- and low-zinc conditions; Mpy was detectable only in the 70S ribosome from the low-zinc culture (Fig. 3B). The difference in Mpy levels between high- and low-zinc 70S ribosomes purified using SDG is consistent with our published study in *M. smegmatis* and *M. tuberculosis*, although zinc-independent abundance of Mpy in the total ribosome collected through 32% sucrose cushion reveals growth-phase-dependent interaction between Mpy and the ribosome, albeit with varied strengths, some of which may not be strong enough to withstand the higher downward force over a longer duration in the SDG-based purification method. However, under specific starving conditions like low-zinc, factors like Mrf further stabilize the Mpy-ribosome complex, rendering it more resistant to physical, and perhaps other, stresses. Moreover, the difference in the Mpy levels between total and fractionated ribosomes in Fig. 3B also highlights the technical limitations of each purification method and the need for new considerations in the approach based on the line of interrogation.

### Mpy-dependent ribosome hibernation leads to amikacin tolerance

**Fig 4:**
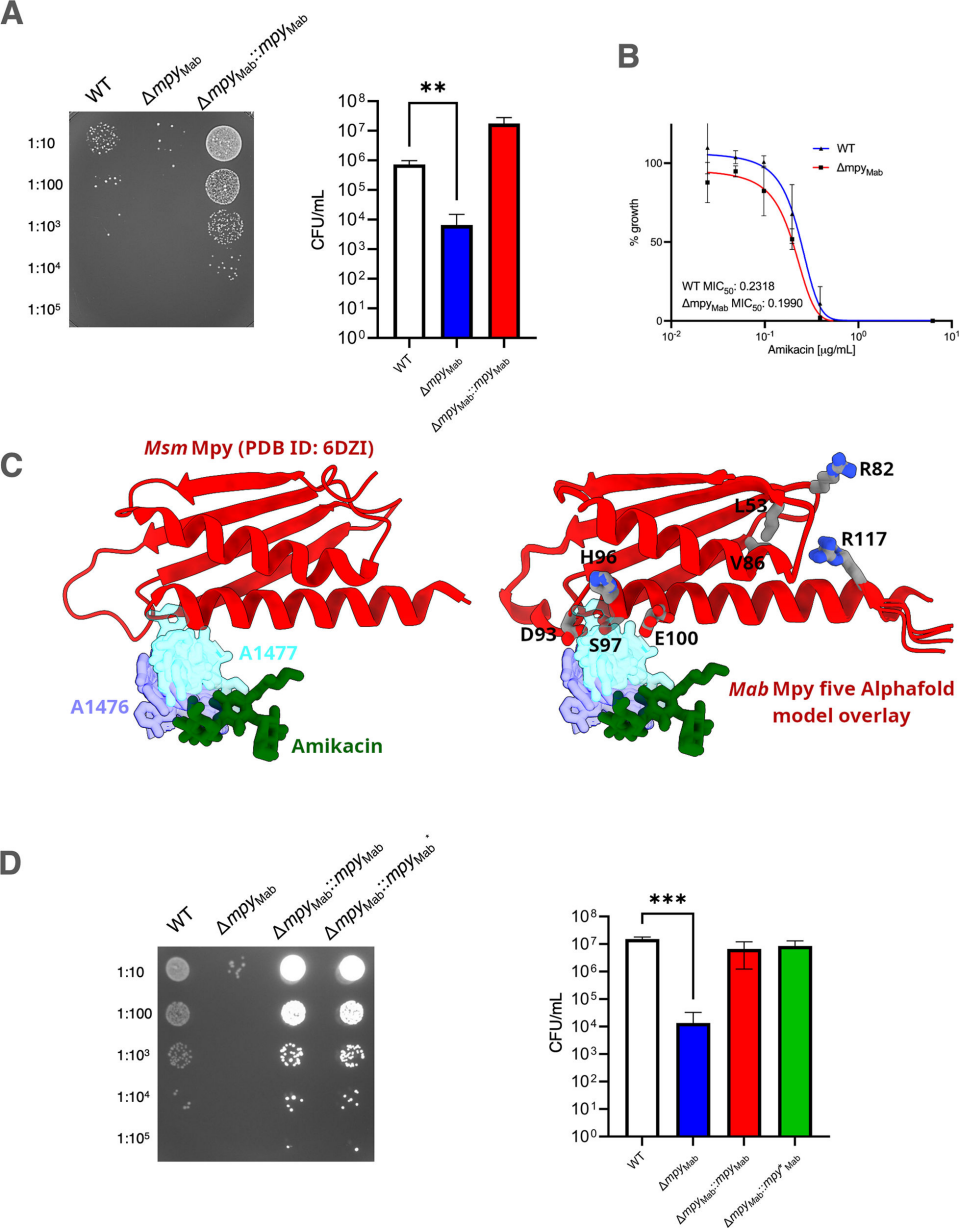
Effect of Mpy on amikacin sensitivity in *M. abscessus*. (**A**) Effect of *mpy*_Mab_ deletion on amikacin sensitivity in *M. abscessus*. Cells of wild-type (WT), Δ*mpy*_Mab_, or Δ*mpy*_mab_::*mpy*_Mab_ cultured in low-zinc Sauton’s medium for 7 days were resuspended in low-zinc PBS (0.05% Tween and 2 μM TPEN) with 40 μg/mL of amikacin for 7 days before enumerating the viable cells. The plot to the right shows mean ± SD CFU/ml from three biologically independent experiments (**P < 0.01, two-tailed Student’s *t*-test). (**B**) MIC of amikacin for WT and Δ*mpy*_Mab_ in low-zinc Sauton’s media, indicating that the Mpy-dependent amikacin tolerance is pronounced under starvation. (**C**) Overlay of Mpy_Mab_ (A1476 (lavender) and A1477 (bright cyan) conformations (*M. smegmatis* numbering) with the bound amikacin conformation (dark green) showing Mpy_Mab_ residues D93, H96, S97, and E100 (gray sticks) that are likely to affect A1476–A1477 motion and influence amikacin binding. Mpy_Mab_ AlphaFold structure (A0A829PTU8) is shown in red ribbons. (**D**) Complementation of amikacin sensitivity in Δ*mpy*_Mab_ by harboring Ala substitutions in D93A, H96A, S97A, and E100A (*mpy*_mab_^*^). Low-zinc (2 μM TPEN) stationary-phase cells of starved wild-type, Δ*mpy*_Mab_, Δ*mpy*_Mab_::*mpy*_Mab_, or Δ*mpy*_Mab_:: *mpy*_Mab_^*^ cells were resuspended in low-zinc PBS (0.05% Tween and 2 μM TPEN) with 40 μg/mL of amikacin for 7 days before enumerating the viable cells. Data in the plot represent mean ± SD from three independent experiments. ***P < 0.001 (unpaired two-tailed Student’s *t*-test).

A relationship between ribosome hibernation and aminoglycoside tolerance has been reported in *E. coli* (25), *L. monocytogenes* (26), *V. cholerae* (27), *M. smegmatis* (13), and *M. tuberculosis* (10). The interference of Mpy with the activities of kanamycin and streptomycin has been predicted from the structure in *M. smegmatis* (13), and Mpy-dependent tolerance to these drugs has been demonstrated in *M. smegmatis* as well as *M. tuberculosis* (10, 13). As expected from our previous observations*,* amikacin exposure in the Δ*mpy*_Mab_ strain led to greater loss of viability by ~2 orders of magnitude compared with the wild-type and complemented strains (Fig. 4A). We note that the amikacin sensitivity of Δ*mpy*_Mab_ was observed when cells were grown to stationary phase in low-zinc Sauton’s medium followed by exposure to amikacin-containing PBS. However, the amikacin minimal inhibitory concentration (MIC) was similar for Δ*mpy*_Mab_ and wild-type strains (Fig. 4B). Given that MIC is determined using actively growing cultures, lack of amikacin sensitivity in a growing culture of Δ*mpy*_Mab_ is consistent with the model that Mpy-dependent antibiotic tolerance is limited to nutrient-starved conditions when Mpy-ribosome interaction is expected to be maximum. In summary, we conclude a role for Mpy in countering amikacin sensitivity in growth-arrested *M. abscessus*.

We also tested the susceptibility of Δ*mpy*_Mab_ to the other clinically relevant ribosome-targeting antibiotics, clarithromycin and tigecycline. Of these, clarithromycin binds to the 50S subunit and hence Mpy binding is not expected to impact on the clarithromycin sensitivity (Fig. S1A). Expectedly, clarithromycin did not result in decreased viability of growth-arrested low-zinc Δ*mpy*_Mab_ cells relative to the wild-type or complemented strain (Fig. S1B).

The tigecycline-binding pocket on the 30S subunit includes h34 (PDB ID: 5J91) in close proximity to the Mpy binding region (13, 28, 29) (Fig. S2A), suggesting that Mpy binding might influence the tigecycline sensitivity. However, we did not observe any Mpy-dependent tigecycline sensitivity (Fig. S2B), demonstrating that tigecycline binding to the ribosome is unaffected by the occupancy of Mpy in the proximal region.

**Fig 5:**
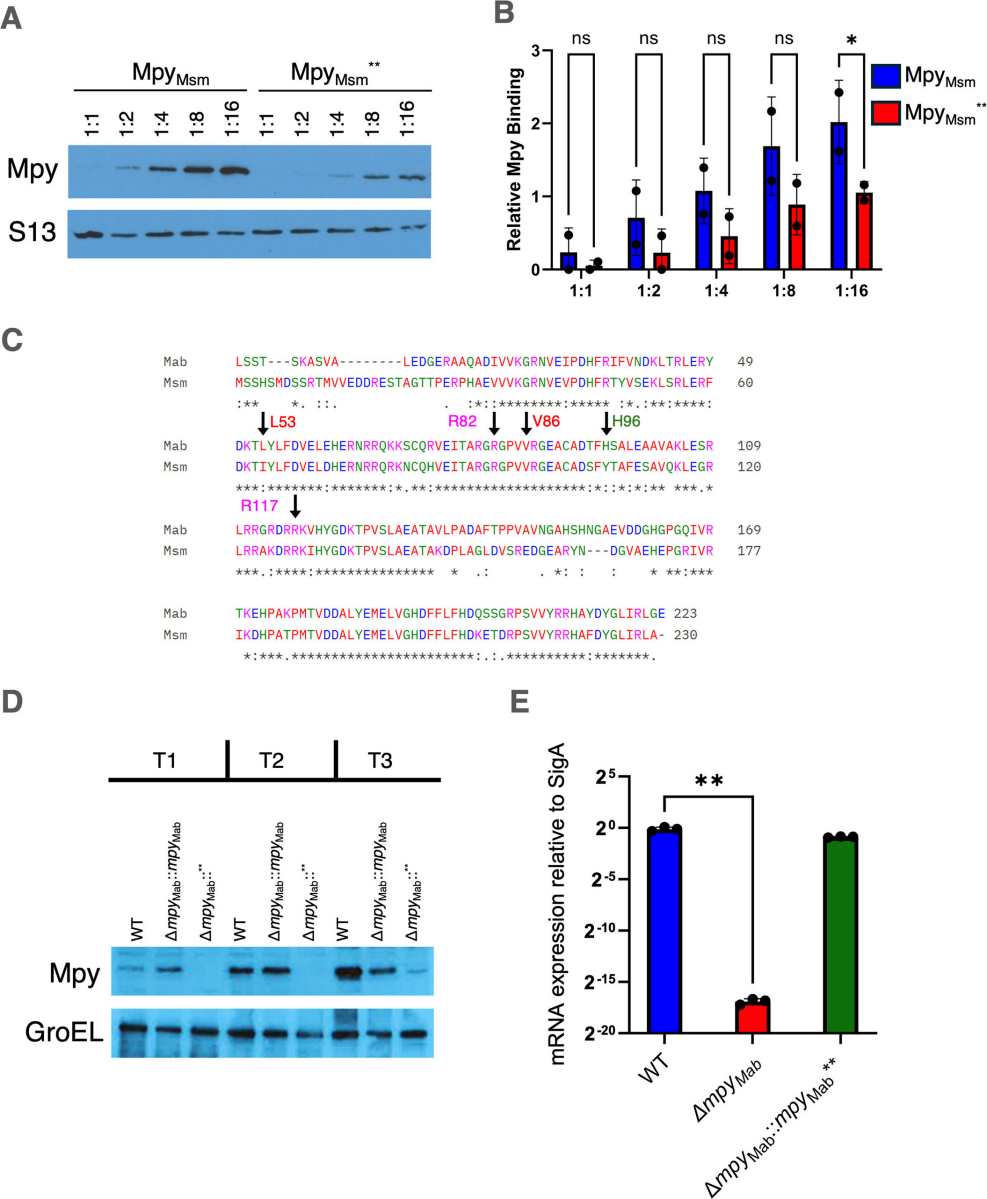
Ribosome-binding residues in *M. abscessus* Mpy are critical for its intracellular stability. (**A and B**) Co-sedimentation of recombinant Mpy_Msm_ or Mpy^**^_Msm_ (harboring Ala substitutions in I64, R93, V97, and R128) with the ribosome purified from low-zinc (1 µM TPEN) stationary-phase cells of *M. smegmatis* Δ*mpy* strain. Ribosome (2.4 pmol) was mixed and incubated with Mpy in the indicated molar ratio and Mpy-bound particles were separated from the free form by ultracentrifugation on a 32% sucrose cushion. The resulting pellets were analyzed for S13 to confirm pelleting of ribosome, and Mpy to test its interaction with the ribosome. Data in panel **B** represent mean ± SD from two independent experiments. *P < 0.05 (two-way ANOVA), ns denotes not-significant. (**C**) Alignment of Mpy_Mab_ and Mpy_Msm_ amino acid sequences, with Mpy_Mab_ residues L53, R82, V86, and R117, which were mutated to alanine in Δ*mpy*_Mab_::*mpy*_Mab_^**^, and H96 are indicated by arrows. (**D**) Levels of Mpy_Mab_ or Mpy _Mab_^**^ in cell lysates from *M. abscessus* wild-type (WT) and its Δ*mpy*_Mab_::*mpy*_Mab_ and Δ*mpy*_Mab_::*mpy*_Mab_^**^ strains, respectively, cultured in low-zinc Sauton’s medium at time points corresponding to Fig. 3. Ten micrograms of lysate was probed for Mpy. GroEL was probed as loading control. (**E**) Mpy mRNA in the indicated strains relative to SigA transcript, determined by RT-qPCR. Data represent mean ± SD of three biologically independent experiments. *P < 0.05 (unpaired two-tailed Student’s *t*-test). ns denotes not-significant.

We next sought to identify the residues in Mpy_Mab_ responsible for interfering with amikacin-ribosome interaction. Binding of 2-deoxystreptamine (2-DOS) aminoglycosides, such as paromomycin, kanamycin, or amikacin to the ribosome results in the flipping out of A1492 and A1493 in *E. coli* (30, 31). Additionally, structures of the bacterial ribosomal decoding site on the 30S subunit complexed with amikacin reveals that amikacin binds in a similar manner to that of its parent compound kanamycin (32, 33). The cryo-EM structure of the Mpy-bound C− ribosome predicts that Mpy residue Y107 in *M. smegmatis* occupies the space needed for A1477 (1492 in *E. coli*) to flip out and interact with the mini-helix between a near-cognate tRNA and the mRNA codon (13). Thus, Y107 was predicted to sterically interfere with the kanamycin–ribosome interaction and thereby reduce kanamycin toxicity in the cell. Alignment of an AlphaFold prediction of Mpy_Mab_ with the cryo-EM Mpy_Msm_ structure suggests highly similar folds in the RNA-binding regions of both the proteins (Fig. S3). However, Y107 is replaced by a histidine (H96) in Mpy_Mab_, while the homologs from multiple other species retain a highly conserved tyrosine at this position (Fig. 5C). Given the substitution of histidine at the conserved Y107 site, we reasoned that H96, and possibly additional residues, could be responsible for reducing the flipping out of A1477 necessary for amikacin resistance. To understand the relationship between the Y107/H96 residue and A1477, we evaluated all conformations of A1476 and A1477 within mycobacterial ribosomal 30S subunits from the available structural data (34–42) (see Table S1). We aligned these structures using their 16S rRNA in the vicinity of the binding site of 2-DOS aminoglycosides, such as kanamycin or amikacin. In the absence of a 2-DOS aminoglycoside, the residues A1476 and A1477 exhibit multiple conformations (Fig. 4C). We then superimposed the conformational states of the A1476 and A1477 residues in *M. smegmatis* onto the AlphaFold models of Mpy_Msm_ (A0QTK6) and Mpy_Mab_ (A0A829PTU8) (Table S1) and identified four residues in *M. abscessus* Mpy (D93, H96, S97, and E100) as closest to A1476 and A1477 (Fig. 4C). However, complementation analysis showed that an Mpy variant containing D93A, H96A, S97A, and E100A substitutions (Δ*mpy*_Mab_::*mpy*_Mab_*) had a similar level of amikacin sensitivity as wild type Mpy_Mab_ (Δ*mpy*_Mab_::*mpy*_Mab_), indicating that D93, H96, S97, and E100 in the *M. abscessus* Mpy are not sufficient to confer amikacin tolerance (Fig. 4D). Perhaps a broader region of the contact surface of Mpy with the ribosome confers amikacin tolerance or the degree of flipping of A1476 and A1477 bases required for amikacin binding is just as compatible with smaller alanine residues at those four positions.

### Ribosome-binding residues are necessary for mpy stability in *M. abscessus*

To further elucidate the molecular basis of Mpy-dependent ribosome hibernation in *M. abscessus*, we asked which residues of Mpy were important for interaction with the ribosome. Analysis of the cryo-EM solved structure of the Mpy-bound C− *M. smegmatis* ribosome (PDB 6DZI) indicated residues I64, R93, V97, and R128 as crucial for binding to the ribosome. A recombinant Mpy_Msm_ containing mutations in each of these residues to alanine (Mpy_Msm_^**^), purified from *E. coli*, exhibited a severe defect in its ability to bind to the ribosome *in vitro* (Fig. 5A and B). We therefore transformed Δ*mpy*_Mab_ with a plasmid carrying *mpy*_Mab_ with alanine substitutions in the residues corresponding to I64, R93, V97, and R128; L53, R82, V86, and R117 (Fig. 5C) (Δ*mpy*_Mab_::*mpy*_Mab_^**^). We assayed for the presence of Mpy_Mab_ in cell lysates from wild-type, Δ*mpy*_Mab_::*mpy*_Mab_ and Δ*mpy*_Mab_::*mpy*_Mab_^**^ cultures from logarithmic (OD_600_ = 0.5), early-stationary (42 h post-inoculum), and late-stationary (7 days post-inoculum) phases. Despite a robust transcription of Mpy_Mab_^**^ , its protein was undetectable at any of the three time points (Fig. 5E), indicating that residues likely involved in the ribosome interaction are also important for the stability of Mpy in the *M. abscessus* cytosol.

## DISCUSSION

In this study, we demonstrate ribosome remodeling and hibernation of the remodeled ribosome in zinc-starved *M. abscessus*, similar to our previous observations in *M. tuberculosis* and *M. smegmatis* (10, 13). Ribosome hibernation not only preserves integrity of the ribosome during growth arrested conditions but also reduces bacterial sensitivity to the frontline aminoglycoside amikacin, highlighting this pathway as a survival strategy in *M. abscessus*.

Models of ribosome hibernation in multiple bacterial species are broadly centered around the paradigm that the process is induced by limiting nutrients during transition into stationary phase (40, 43–49), although precise identity of the specific nutrient, limitation of which induces ribosome hibernation in the stationary phase, remains unknown. In our previous studies, we observed that *M. smegmatis* and *M. tuberculosis,* when cultured in medium with limiting amounts of free zinc exhibit high levels of Mpy-bound ribosomes, demonstrating that zinc limitation is a condition for inducing Mpy interaction with the ribosome. However, Mpy-dependent ribosome hibernation induced by limitation of other macronutrients is unresolved. Here, we systematically compared the levels of Mpy-bound ribosomes in *M. abscessus* during late-stationary phase under high- and low-zinc conditions using distinct purification methods. While sedimentation through a sucrose cushion does not resolve individual ribosomal species and can preserve relatively weak ligand–ribosome interactions, SDG fractionation on the other hand separates ribosomal species and is a more stringent method for screening ribosome-protein interactions (50). Our finding that Mpy is detectable in total ribosomes purified using sucrose cushion from late-stationary phase cultures under both high- and low-zinc conditions, but undetectable in high-zinc upon fractionation in SDG, indicates that Mpy interacts with the ribosome in a growth-phase dependent manner, independent of zinc availability. The selective retention of Mpy on 70S ribosomes under low-zinc conditions following SDG fractionation however reveals distinct strength of interaction under different conditions, suggesting that specific conditions like zinc limitation could further stabilize Mpy-ribosome interaction, likely through the involvement of specialized factors like Mrf (10, 13). Thus, it is reasonable to propose that ribosome purification using sucrose cushion is a more appropriate method for investigating growth-phase dependent ribosome hibernation and its physiological implication. We further propose such studies to be performed in low-zinc *in vitro* cultures, because low-zinc environment sufficient to induce ribosome remodeling is prevalent in infected host lungs (11–13).

A key consequence of ribosome hibernation is altered antibiotic susceptibility. We show that loss of Mpy sensitizes *M. abscessus* to amikacin but not to clarithromycin or tigecycline under growth-arrested conditions. Our results extend previous observations in multiple bacterial species, including *M. smegmatis* and *M. tuberculosis*, and establish Mpy as an aminoglycoside tolerance factor in *M. abscessus*. Despite their structural proximity to the flipped conformations of A1476 and A1477, alanine substitution of residues D93, H96 (Y107 in *M. smegmatis*), S97, and E100 did not abolish Mpy-dependent amikacin sensitivity. This result suggests that the likely inhibition of amikacin-ribosome interaction by Mpy may not arise from mere steric hindrance by the conserved tyrosine observed in other species, but rather from a broader region of interaction between Mpy and the ribosome decoding center.

Strikingly, residues expected to be required for ribosome binding are also essential for Mpy protein stability in *M. abscessus*. Alanine substitution of residues implicated in ribosome interaction (L53, R82, V86, and R117) resulted in undetectable level of Mpy_Mab_** in cell lysates, including those from logarithmic-phase cultures, where Mpy is expected to be largely ribosome-free. In contrast, wild-type Mpy remained stable under these conditions, indicating that these residues confer intrinsic stability to Mpy in mycobacteria, thereby confounding our attempts to investigate the role of these residues in Mpy-ribosome interaction and subsequent amikacin tolerance. The intrinsic relationship between stability of Mpy and its ribosome-binding properties suggests that Mpy has evolved to be an obligate ribosome-binding protein. Consequently, perturbation of this region abolishes Mpy activity, likely by destabilizing the protein.

The *in vivo* relevance of zinc-responsive ribosome remodeling and hibernation is supported by evidence that the human lung constitutes a zinc-limiting environment for bacterial pathogens. Zinc levels in sputa expectorated from patients with tuberculosis or cystic fibrosis are sufficiently low to induce established zinc starvation responses in *M. tuberculosis* and *Pseudomonas aeruginosa*, respectively (11, 51). These observations indicate that pathogenic mycobacteria are likely to encounter zinc limitation during infection, providing a physiological context in which C− ribosome assembly and Mpy-mediated hibernation of the C- ribosome would be favored. Under such conditions, ribosome hibernation may contribute to persistence by stabilizing non-translating ribosomes during growth arrest while simultaneously reducing susceptibility to clinically relevant ribosome-targeting antibiotics such as amikacin. Thus, zinc limitation in the airway environment may represent an important determinant of the ribosome state and antibiotic tolerance in pathogenic mycobacteria *in vivo*.

In summary, Mpy functions as a conserved hibernation-promoting factor in mycobacteria, including *M. abscessus*. By stabilizing ribosomes in a translationally inactive state and conferring tolerance to aminoglycosides under low-zinc conditions, Mpy emerges as a key drug target for simplifying therapeutic interventions against infections by mycobacteria, including *M. abscessus*.

## MATERIALS AND METHODS

### Bacterial strains, plasmids, and growth conditions

*Mycobacterium abscessus* ATCC19977 was used as the wild-type strain throughout this study. An isogenic, apramycin resistant Δ*mpy*_Mab_ mutant was generated as described below. Unless otherwise stated, *M. abscessus* strains were grown at 37°C in Middlebrook 7H9 broth supplemented with 10% OADC and 0.05% Tween-80. Complemented strains (Δ*mpy*_Mab:_:*mpy*_Mab_, Δ*mpy*_Mab_::*mpy*_Mab_*, and Δ*mpy*_Mab:_:*mpy*_Mab_**) were maintained in the same medium supplemented with 40 µg/mL kanamycin. Solid media for *M. abscessus* consisted of Middlebrook 7H10 agar supplemented with 10% OADC. For high-zinc and low-zinc conditions, primary cultures were washed three times in Sauton’s medium containing 0.05% Tween-80 and supplemented with either 1 mM ZnSO₄ (high-zinc) or 2 µM TPEN (low-zinc). Cultures were back-diluted to an OD_600_ of 0.1 and incubated with shaking at 37°C for the indicated time points. TPEN was added at the time of inoculation. Zinc concentrations were chosen based on previously published work (10), and zinc limitation was operationally defined by TPEN treatment and validated by immunoblot of C− ribosomal protein paralogs. *Mycobacterium smegmatis* cultures were grown on Middlebrook 7H10 agar supplemented with 10% ADC. *Escherichia coli* DH5α derivatives were grown in LB broth or agar at 37°C with appropriate antibiotics.

### Construction of mutant strains and plasmids

All recombinant plasmids, bacterial strains, and oligonucleotides used in this study are listed in Tables S1 and S2. The Δ*mpy*_Mab_ strain was generated using a PCR-based modified recombineering strategy adapted for *M. abscessus* (52, 53). Targeting arms flanking *mpy*_Mab_ were amplified from ATCC19977 genomic DNA using primers listed in Table S3. Each homology arm of the allelic exchange substrate (AES) was 424 bp in length. The left arm was amplified with distal and proximal primers containing SpeI and XbaI restriction sites, respectively, while the right arm was amplified with distal and proximal primers containing HindIII and SpeI sites, respectively. The left flanking arm was ligated into pYUB-Apramycin following digestion with subsp. and XbaI. The resulting construct was digested with HindIII and SpeI and ligated with the right flanking arm. The proximal primer for each arm had a tail complementary to opposite ends of a PCR-amplified apramycin resistance cassette. The apramycin resistance cassette consists of an upstream universal priming site (UPS), a *loxP* site, an T7 promoter and apramycin resistance gene, a second *loxP* site, and another unique priming site. pYUB-apramycin containing the UPS-tailed arms was transformed into *E. coli*, and the genotypes of apramycin-resistant colonies were confirmed by PCR. pYUB-apramycin containing the UPS-tailed arms with the UPS-tailed apramycin-resistance cassette was electroporated into recombineering-proficient *M. abscessus* cells containing the recombineering plasmid (pJV53-sacB) (53). The genotypes of apramycin-resistant colonies were confirmed by PCR. pJV53-sacB was removed by plating the cells on 7H10OADC with 15% sucrose.

All complementing plasmids were constructed in an L5-attP based integrative vector backbone, pEM2 (Kan^r^), which was constructed in a pMH94 backbone. p*mpy*_Mab_* and p*mpy*_Mab_** were created by introducing fragments from Twist Bioscience which contained KpnI and XbaI restriction sites into pEM2. Sequences of these fragments are noted in Table S2.

Mutations were sequence-confirmed, and the plasmids p*mpy*_Mab_, p*mpy*_Mab_*, and *mpy*_Mab_** were electroporated into *M. abscessus* ATCC19977 to generate Δ*mpy*_Mab_::*mpy*_Mab_, Δ*mpy*_Mab_::*mpy*_Mab_*, and Δ*mpy*_Mab_::*mpy*_Mab_**, respectively.

### Real-time quantitative-PCR (RT-qPCR)

One-milliliter culture of the indicated strains of *M. abscessus* obtained from the specified conditions was pelleted and resuspended in 500 μL Qiagen RLT Buffer (QIAGEN Inc.), and an equal volume of BioSpec zirconia/silica beads was added. The resulting suspension was beaten at 4°C using a Bead-beater (Biospec Products; eight 45s cycles with 30s intervals) for cell lysate preparation. Total RNA was purified from the resulting lysates using a Qiagen RNeasy Mini Kit (QIAGEN Inc.) according to the manufacturer’s instructions. *M. abscessus* RNA was then DNase-treated using TURBO DNase (Thermo Scientific) according to the manufacturer’s instructions. cDNA was prepared from the resulting DNA-free RNA using a Maxima First Strand cDNA Synthesis Kit according to the manufacturer’s instructions. Real-time PCR was performed on an ABI 7000 instrument according to the manufacturer’s instructions. 10 ng of cDNA was mixed with 1 μL of 5 μM primers and 10 μL of SYBR Green Master Mix in a final volume of 20 μL. PCR was performed under the following conditions: 95°C for 10 min, followed by 40 cycles of 95°C for 10 s and 60°C for 1 min. For each set of experiments, an endogenous control with *M. abscessus sigA*-specific primers, as well as no-template controls, were set up for normalization. RNA that did not undergo reverse transcription was included to test for genomic DNA contamination prior to setting up the RT-qPCR.

### Ribosome purification

Broadly, ribosome purification was performed, as previously reported (54). Indicated strains of *M. abscessus* were cultured in 500 mL of Sauton’s medium +0.05% Tween-80 supplemented with either 1 mM ZnSO4 for high-zinc culture or 2 μM of TPEN for low-zinc culture for the indicated time points. The cells were collected by centrifugation at 8,000 rpm for 20 min at 4°C utilizing a Thermo Scientific Fiberlite F12−6× 500 fixed-angle rotor and stored frozen at −80°C until use. Cells were thawed and spread into a thin layer coating the walls, flash-frozen in liquid nitrogen, scraped, and pulverized using a mixer mill (Retsch MM400), 12 cycles each of 3 min at a frequency of 15 Hz.

Milled cellular material was subsequently resuspended in 15 mL of cold HMA-10/30 buffer (10 mM MgCl_2_, 30 mM NH_4_Cl, 20 mM HEPES-K at pH 7.5, and 5 mM β-mercaptoethanol) and was centrifuged at 15,000 rpm for 30 min at 4°C in a Thermo Scientific F21−8 × 50y fixed-angle rotor. Then, 500-µL aliquots from the resultant supernatants were set aside as crude cell lysate. Remaining supernatants were transferred into Beckman PC ultracentrifuge tubes (Beckman 355,618) and subjected to centrifugation at 42,800 rpm for 2 h and 15 min at 4°C utilizing a Ty70Ti, Type 70 Ti Fixed-Angle Titanium Rotor (339,722) from Beckman. The supernatants from the ultracentrifugation were discarded, and the resultant pellets were soaked in 1 mL of HMA-10/30 overnight.

The soaked pellets were subsequently resuspended and homogenized in 2 mL of HMA-10/30 buffer for 20 min on ice and were subsequently diluted with another 2 mL of HMA-10/30 buffer before treating with 3 units/mL TURBO DNase (2 U/μL) (Invitrogen, #AM2238) for 1 h on ice. Unless otherwise indicated, 4 mL of high-salt wash buffer (HMA 10/600 buffer: 10 mM MgCl_2_, 600 mM NH_4_Cl, 20 mM HEPES-K pH 7.5, 5 mM β-mercaptoethanol) was added to the DNase-treated homogenates and incubated at 4°C for 2 h to remove weakly associated proteins. The homogenates were subsequently centrifuged at 13,000 rpm for 30 min at 4°C. The resulting supernatants were then carefully transferred into Beckman PC ultracentrifuge tubes and centrifuged at a velocity of 42,800 rpm for a period of 2 h and 15 min at 4°C utilizing a Ty70Ti rotor. The supernatants were discarded, and the resultant pellets containing the crude ribosomes were incubated in 500 μL of HMA-10/30 buffer overnight. The pellets were resuspended in 1 mL of HMA-10/30 buffer. To obtain crude ribosome, the samples were centrifuged at 13,000 rpm for a duration of 15 min at 4°C using a benchtop centrifuge (Eppendorf 5415D). The crude ribosomes were quantified by measuring absorbance at 260 nm.

A total of 600 pmol of crude ribosome in up to 500 μL of HMA-10 buffer was meticulously layered atop a 40 mL sucrose gradient ranging from 10% to 40% in HMA-10/30 buffer and centrifuged for a duration of 16 h at 24,000 rpm in a Beckman SW28 rotor. The resultant gradients were subsequently fractionated with a Brandel density gradient fractionation system to isolate individual ribosomal subunits. Fractions associated with the 70S ribosomal complexes were collected individually, subsequently diluted with HMA-10/30 buffer, and ultracentrifuged at 42,800 rpm for 16 h at 4°C utilizing a Beckman rotor Ty70Ti. The resultant pellets were incubated overnight in 500 μL of HMA-10/30 buffer and then further diluted with an additional 500 μL of HMA-10/30 buffer, achieving a final volume of 1 mL that primarily constituted purified 70S ribosomes. The concentrations of the ribosome samples were determined by measuring A_260nm_. The samples were stored at −80°C for future applications.

In the indicated experiments, some crude ribosome samples were purified from a 7-day culture on a 32% sucrose cushion using ultracentrifugation at 45,000 RPM for a duration of 16 h in a Ty70Ti rotor. The resultant supernatant was discarded, leaving the pellet containing a mixture of 30S, 50S, and 70S ribosomes. The resultant pellets were resuspended in HMA-10/30 buffer for analysis.

### Growth curve and sampling for systematic fractionation analysis

Wild-type *M. abscessus* ATCC19977 was cultured at 37°C with shaking in Sauton’s medium supplemented with 0.05% Tween-80 under either high- or low-zinc conditions. Cultures were sampled at three growth stages: logarithmic phase (OD₆₀₀ = 0.5), early stationary phase (42-h post-inoculation), and late stationary phase (7 days post-inoculation). At each time point, cells were harvested, and crude ribosomes were prepared as described above. Crude ribosome preparations were then processed by ultracentrifugation either on a 32% sucrose cushion or a 10%–40% SDG, as described above, to compare Mpy association across growth stages and zinc conditions.

### Immunoblotting

For analysis of Mpy and GroEL in cell lysates, *M. abscessus* cells were cultured at 37°C with shaking in high- or low-zinc Sauton’s medium supplemented with 0.05% Tween-80. Cultures were harvested, resuspended in 1 mM phenylmethylsulfonyl fluoride, 0.1% Triton X-100, 1 mM ethylenediaminetetraacetic acid (EDTA), and 0.05% Tween 80 in PBS, and lysed in a bead beater. Protein concentrations in cell lysates were determined by Bradford assay. Proteins equivalent to 20 μg from each lysate were mixed with 4 μL of 4× SDS loading buffer (16 μL total volume) and heated for 10 min at 95°C. Equal amounts of denatured proteins from each lysate were resolved in 12% SDS-PAGE gel (for GroEL and Mpy), and proteins were transferred to PVDF membranes utilizing the BioRad Trans-Blot SD Semi-Dry Transfer Cell for a period of 70 min at a current of 105 mA.

For analysis of Mpy and S13 in the ribosome, 2.4 pmol of 70S ribosomes or total ribosomes derived from specified strains and time points were combined with a 4× SDS loading buffer to achieve a final volume of 12 μL and heated for 10 min at 95°C. Subsequently, the samples were separated using a 15% SDS-PAGE gel and subsequently transferred to PVDF membranes using the BioRad Trans-Blot SD Semi-Dry Transfer Cell for 70 min at 105 mA.

Resultant PVDF membranes were incubated with 5% nonfat milk in TBST (10 mM Tris and 150 mM NaCl with 0.05% Tween-20 detergent) overnight, followed by four washes with TBST, and subsequently were incubated for 1 h with specified antibodies: S13 (Developmental Studies Hybridoma Bank, in mouse, at a dilution of 1:100); S14_C−_ (in-house, in rabbit, at a dilution of 1:2,000); L31_C+_ (in-house, in rabbit, at a dilution of 1:800), GroEL (Enzo, in rabbit, at a dilution of 1:5,000), or Mpy (in-house, in rabbit, at a dilution of 1:5,000). All primary antibody solutions were prepared in 1% nonfat milk in TBST except for S13, which was prepared in TBST. The membranes were developed with ECL chemiluminescent substrate for the detection of horseradish peroxidase reagent (Thermo Scientific, #PI32106) and subsequently exposed to chemiluminescence films. These films were thereafter developed and scanned for visualizing and quantifying protein abundance.

### *In vitro* transcription/translation assay

The assay was performed using PURExpress ΔRibosome Kit (New England BioLabs Inc.), as described previously (10). Briefly, in a total volume of 5 μL, 18 ng of a synthetic DNA template, in which the T7 promoter linked with a Shine-Dalgarno sequence and placed upstream of the Nano-Luc reporter coding sequence was mixed with 2.0 μL of Solution A and 0.6 μL of Factor Mix from the kit and 12.5 nM of *M. abscessus* ribosomes. Following a 1-h incubation period at 37°C, 2 μL of the reaction mixture was combined with 18 μL of H_2_O, 20 μL of the luciferase assay buffer, and 0.4 μL of the luciferin substrate (Nano-Glo Luciferase Assay system, Promega Inc.). After a 5-min incubation at ambient temperature, the relative luminescence units (RLUs) generated by the translated luciferase were quantified in accordance with the manufacturer’s specifications using a Veritas Microplate Luminometer (Turner Biosystems).

### Antibiotic susceptibility

Minimum inhibitory concentrations (MICs) of amikacin for wild-type *M. abscessus* ATCC19977 and Δ*mpy*_Mab_ were determined by inoculation of 0.1 OD_600_ into Middlebrook 7H9 broth supplemented with 10% OADC and 0.05% Tween-80 grown at 37°C in a 200 rpm shaker for 24 h. Cultures were then washed three times in Sauton’s media without zinc, and pellets were finally resuspended in Sauton’s media with 2 µM TPEN. The cell suspensions were dispensed in a series of tubes with twofold serially diluted amikacin. For serial dilution, a 10 mg/mL stock of amikacin was prepared and subsequently added to the initial tube to a final concentration of 6.25 µg/mL, after which twofold dilution series was prepared by sequentially diluting the drug concentration into half by mixing equal volume of preceding drug-containing medium with the next drug-free medium. Three biological replicates of wild-type *M. abscessus* ATCC19977 and Δ*mpy*_Mab_ were inoculated into the dilution series at 0.02 OD_600_. Growth inhibition was measured using a ThermoFisher Genesys 20 spectrophotometer after 48 h of growth in low-zinc conditions. A no antibiotic control was added to determine percent growth. A tube with un-inoculated media was used as blank. MICs_50_ were calculated by nonlinear regression using GraphPad.

For bactericidal assay*, M. abscessus* wild-type ATCC19977, Δ*mpy*_Mab_::*mpy*_Mab_, Δ*mpy*_Mab_::*mpy*_Mab_*, and Δ*mpy*_Mab_::*mpy*_Mab_** were inoculated at an OD_600_ of 0.1 in 50 mL of Sauton’s media with 2 μM TPEN. Cultures were incubated at 37°C for 7 days. Cells were then harvested and washed three times in 50 mL of PBS + 0.05% tyloxapol with 2 μM TPEN. Cells were diluted to an OD_600_ of 0.8 in 5 mL of 0.05% tyloxapol with 2 μM TPEN containing clarithromycin (8 μg/mL), tigecycline (2 μg/mL), or amikacin (40 μg/mL). A drug-free culture was used as control. At regular intervals, serial dilutions were plated on Middlebrook 7H10 agar with 0.5% Glycerol and 10% OADC, and incubated for 3 days before counting the colony-forming units (CFUs). Three biological replicates were analyzed per strain, and statistical significance was assessed using unpaired two-tailed Student’s *t*-test.

### *In vitro* ribosome-binding assay

Purified *M. smegmatis* crude ribosomes (2.4 pmol) from low-zinc cultures were incubated with the indicated amounts of recombinant wild-type Mpy_Msm_ or Mpy_Msm_ harboring Ala substitutions in I64, R93, V97, and R128 (Mpy_Msm_**) in HMA-10/30 buffer at 37°C for 1 h. Following incubation, samples were subjected to ultracentrifugation through a 32% sucrose cushion prepared in HMA-10/30 buffer for 3 h at 4°C using a Beckman TLA-100.3 rotor. After centrifugation, the supernatant was removed, and the pellet was resuspended in HMA-10/30 buffer. Pelleted material was analyzed by 12% SDS–PAGE, followed by immunoblotting to assess co-sedimentation of Mpy with ribosomes.

### Structural modeling

All structures of *M. smegmatis* ribosomes in the RCSB protein databank were obtained and aligned using their 16S RNA chain with the matchmaker module within ChimeraX (55). The structure for the Mpy-bound C− *M. smegmatis* 70S ribosome (PDB 6DZI) was used for Mpy placement. The structure of *M. tuberculosis* 50S ribosome subunit bound to clarithromycin (PDB 7F0D) (56), the structure of the *T. thermophilus* 70S ribosome bound to tigecycline (PDB 5J91) (57), and the structure of the complex between amikacin and the ribosome (PDB 6YPU, 8EV6, 8SYL) (58, 59) were aligned and analyzed in ChimeraX v1.9 (13, 55, 60).

### Protein sequence alignment

Structural alignments were performed using ChimeraX v1.9. Hibernation promotion factors (HPFs) from multiple bacterial species; *M. smegmatis* 3935, *M. abscessus* Mpy*, M. smegmatis* Mpy, *M. tuberculosis* Mpy*, B. subtilis* long HPF*, S. aureus* long HPF, *L. lactis* long HPF, *E. coli* short HPF, and *E. coli* YfiA were aligned in Clustal Omega.

## Data Availability

All data relevant to the study are presented within the paper and supplemental figures and tables.

## SUPPLEMENTAL MATERIAL

Supplemental figures and tables spectrum.00378-26-s0001.pdf Figures S1 to S3 and Tables S1 to S3.

ASM does not own the copyrights to Supplemental Material that may be linked to, or accessed through, an article. The authors have granted ASM a non-exclusive, world-wide license to publish the Supplemental Material files. Please contact the corresponding author directly for reuse.
