# Supplementary material for: Ribosome hibernation in zinc-starved Mycobacterium abscessus confers amikacin tolerance
Source: Microbiol Spectr. 2026 Jul 6;14(8):e00378-26. doi: 10.1128/spectrum.00378-26 (PMC13435785; doi:10.1128/spectrum.00378-26)
Supplement: Supplementary file 1 [file spectrum.00378-26-s0001.pdf]

Supplementary Figure 1

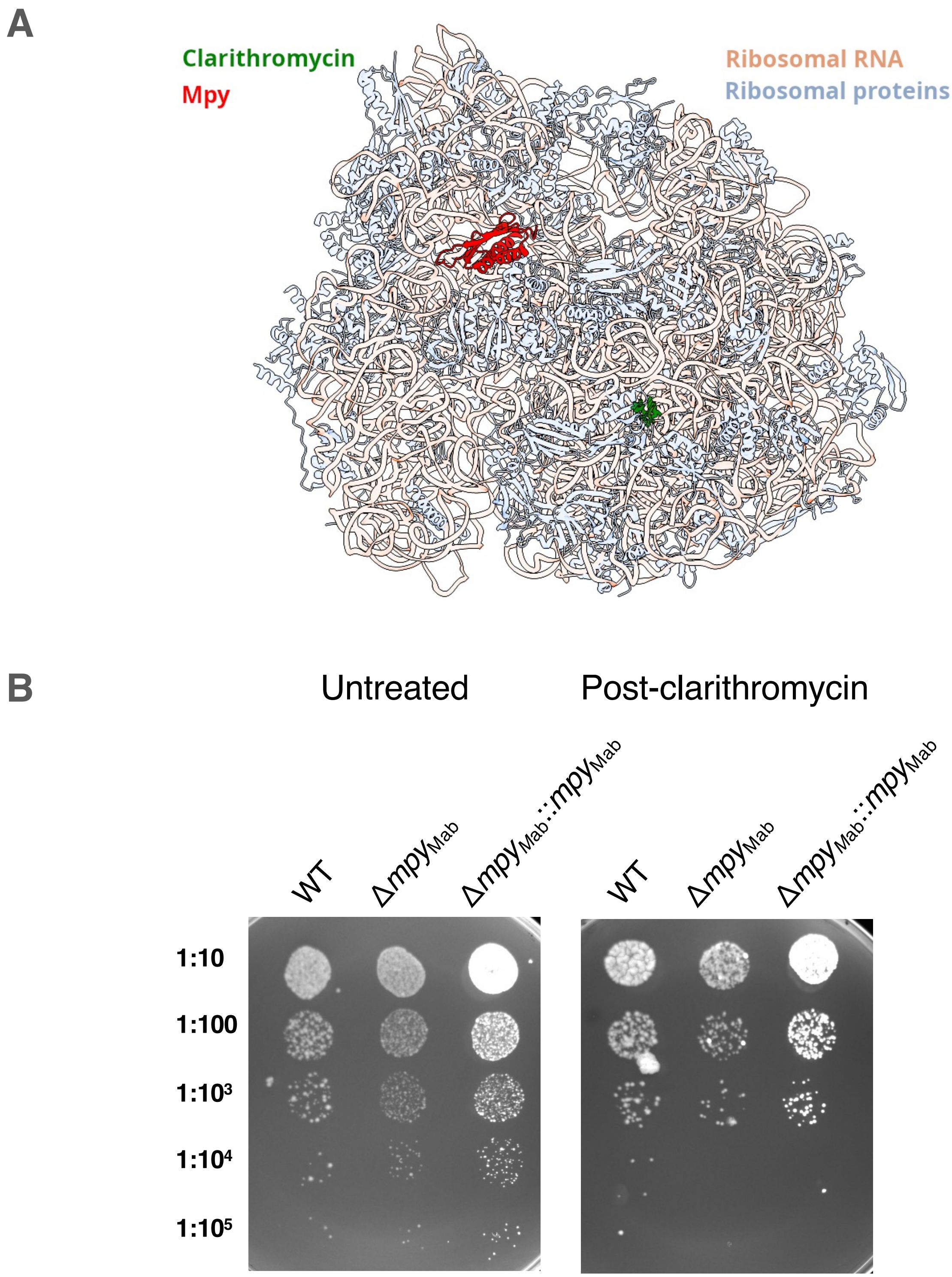

Supplementary Figure 1. Sensitivity of an Mpy mutant of *M. abscessus* to clarithromycin. A. Alignment of Mpy-bound C- *M. smegmatis* 70S ribosome (PDB 6DZI) with structure of *Mycobacterium tuberculosis* 50S ribosome subunit bound with clarithromycin (PDB 7F0D). Mpy (red) is shown on the *M. smegmatis* 16S rRNA and clarithromycin (dark green) is shown on the 23S rRNA. rRNA is shown in pale yellow. **B.** Cells of wild-type, Δmpy_{Mab}, or Δmpy_{Mab}::mpy_{Mab} cultured in low-zinc Sauton's medium for 7 days were resuspended in low-zinc PBS (.05% Tween & 2μM TPEN) with 8 μg/mL of clarithromycin for 7 days before enumerating the viable cells.

Supplementary Figure 2

A

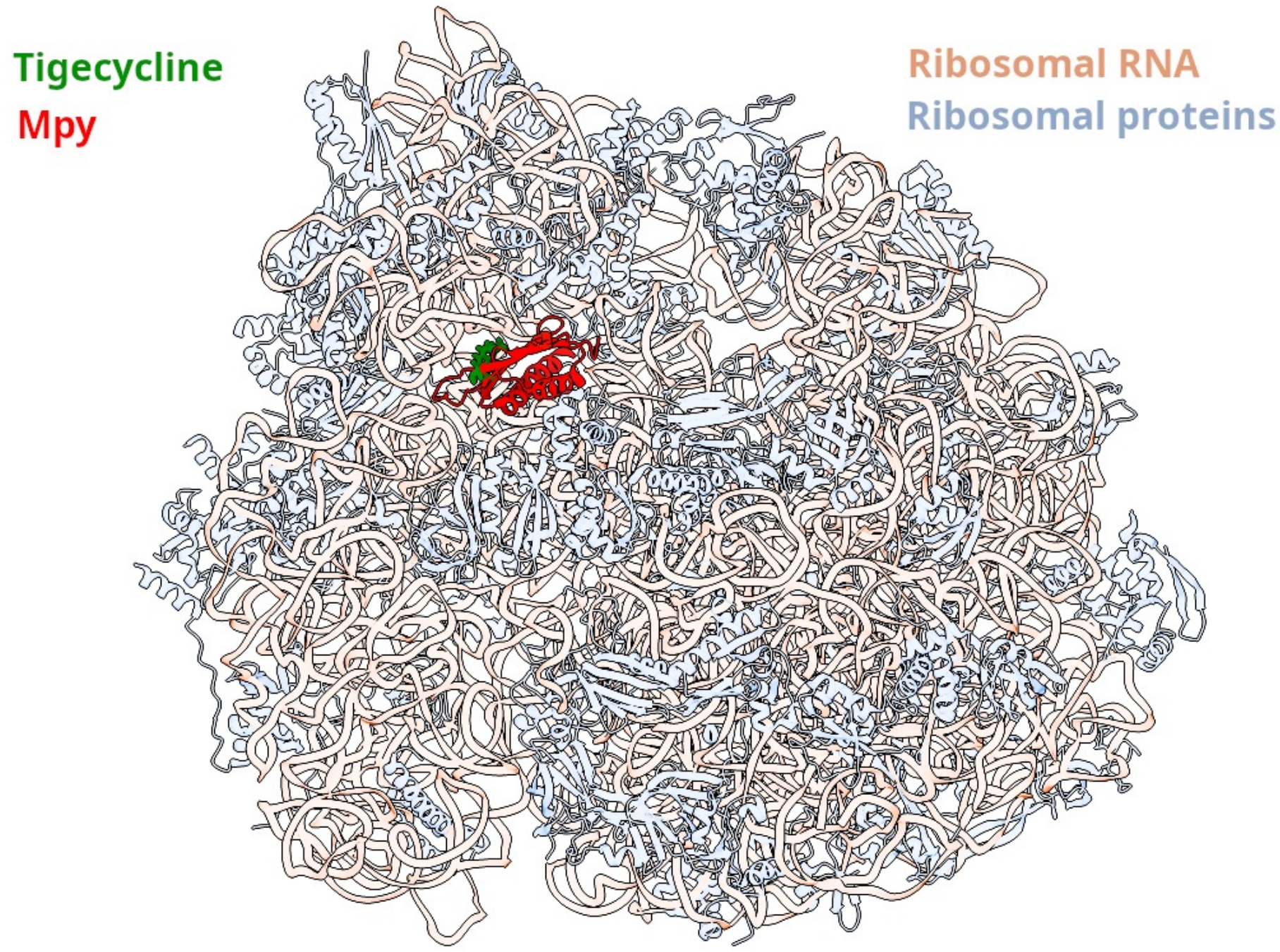

B

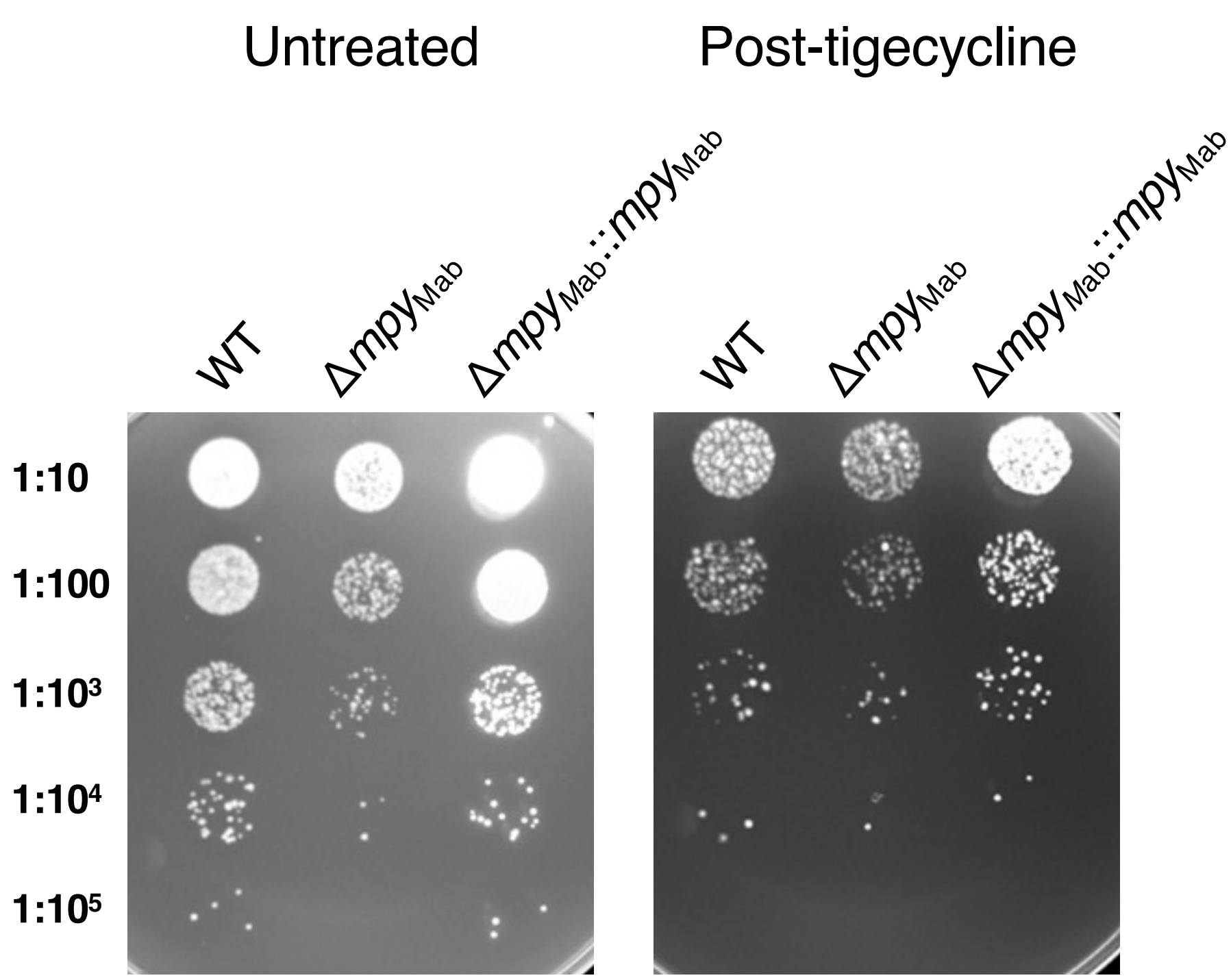

Supplementary Figure 2. Sensitivity of an Mpy mutant of *M. abscessus* to tigecycline. A. Alignment of Mpy-bound C- *M. smegmatis* 70S ribosome (PDB 6DZI) with the *E. coli* 70S ribosome bound with tigecycline (PDB 5J91). Mpy (red) is shown on the *M. smegmatis* 16S rRNA and tigecycline (dark green) is shown adjacent to helix 34 (h34) of the 16S rRNA overlapping the A-site tRNA anticodon-stem loop binding site from *E. coli*. rRNA is shown in pale yellow. **B.** Cells of wild-type, Δmpy_{Mab}, or Δmpy_{Mab}::mpy_{Mab} cultured in low-zinc Sauton's medium for 7 days were resuspended in low-zinc PBS (.05% Tween & 2μM TPEN) with 2 μg/mL of tigecycline for 7 days before enumerating the viable cells.

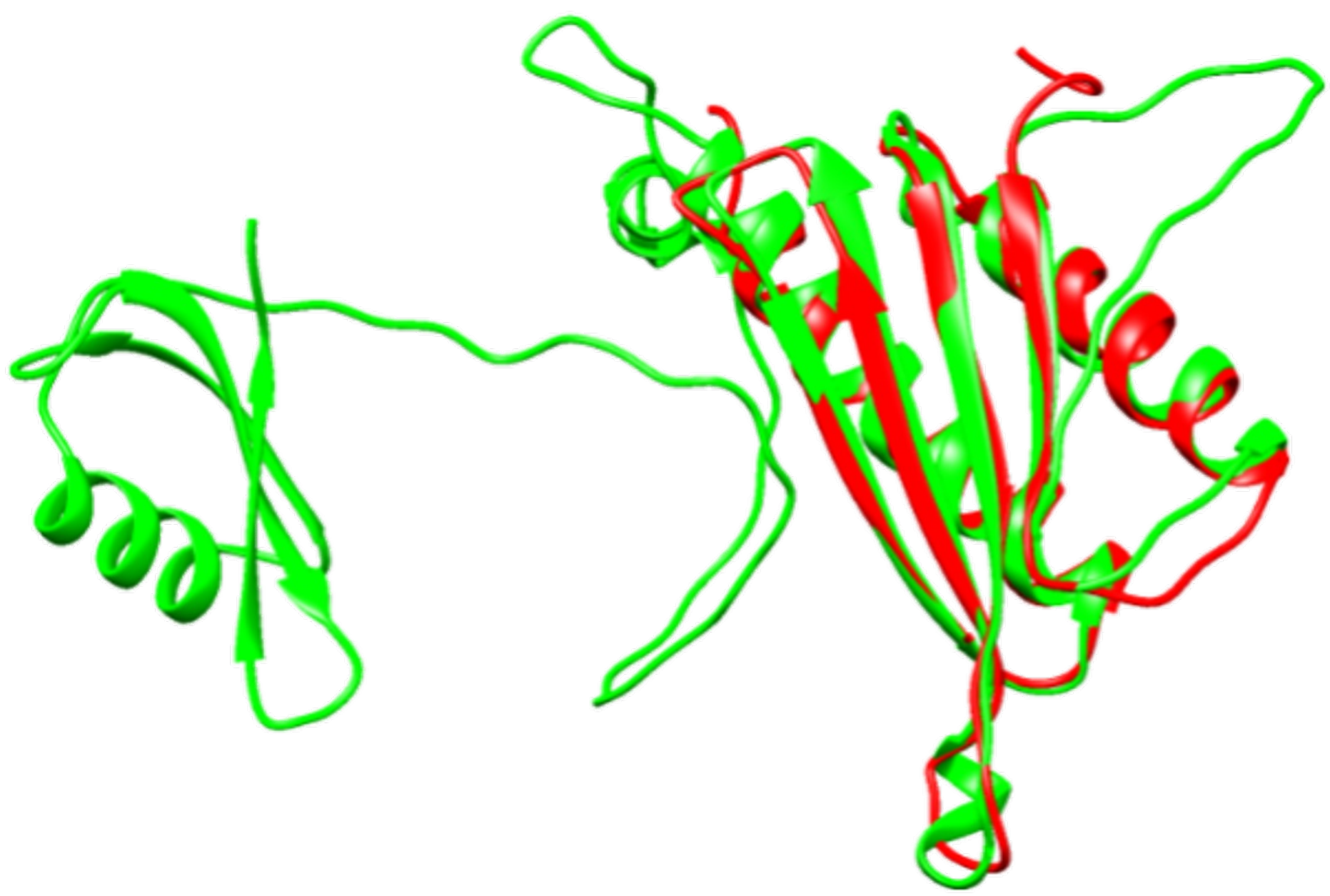

Supplementary Figure 3. Alignment of Alphafold model of *M. abscessus* Mpy (green) with cryo-EM structure of *M. smegmatis* Mpy (6DZI (red), the C-terminal region of which is yet to be resolved.

Table S1. ***Mycobacterium smegmatis*** **ribosome structures used for analysis of A1476 and A1477 flipping.**

Resolution (Å)	PDB ID	16S rRNA Chain ID	Notes	Reference (see main text)
2.0	9N5T	BA	MK-7762 bound	(34)
2.5	9B1Y	A	Wild-type (WT) strain	unpublished
2.5	9B24	A	WT strain gidB mutant	unpublished
2.8	8FR8	a	translation initiation complex with Rsh	(35)
2.8	8WHX	a	RafH	(36)
3.0	8V9L	a	*Msm* Balon and *Msm* EF-Tu(GDP)	(37)
3.1	9B1X	A	HWS19 strain gidB mutant	unpublished
3.1	8V9J	a	*Msm* Balon	(37)
3.1	8V9K	a	*Mtu* Balon	(37)
3.2	9E0P	a	methylated	(38)
3.2	9E0N	a	unmethylated	(38)
3.3	8VIO	a	HflX	(39)
3.3	9B1W	A	HWS19 strain WT	unpublished
3.3	5O61	BA	P-tRNA	
3.4	5ZEB	a	P/P state	(40)
3.4	5ZEP	a	hibernating 70S	(40)
3.5	6DZI	h	hibernating C-minus 70S with Mpy	(13)
3.5	8WI7	a	bS1 and RafH	(36)

3.5	8WIB	a	E-tRNA and RafH	(36)
4.7	9K0Z	A	EF-G2 bound 70S	(41)

Msm – Mycobacterium smegmatis, *Mtu – Mycobacterium tuberculosis*.

Table S2: List of plasmids and bacterial strains used in this study.

Plasmid	Remarks	Reference (see main text)
pMH94	L5-attP-based integrative vector for mycobacteria; kanr	(13)
pYUB-Apramycin	Cosmid vector, Apr	This Study
p*mpy*$_{Mab}$	Mab_3583c with 500bp upstream in XbaI site of pEM2	This Study
p*mpy*$_{Mab}$*	Mab_3583c with 500 base pairs upstream, with the mutations D93A, H96A, S97A, and E100A in pEM2	This Study
p*mpy*$_{Mab}$**	Mab_3583c with 500 base pairs upstream, with the mutations L53A, R82A, V86A, and R117A in pEM2	This Study
prMpy$_{Msm}$		(13)
prMpy$^{**}_{Msm}$		This Study
**Bacterial Strains**		
M. abscessus ATCC19977 (Mab)	*M. abscessus* as parent wild-type	(60)
MabΔ*mpy*$_{Mpy}$	ΔMAB_3583c in *M. abscessus* ATCC19977, apr^{r}	This Study
MabΔ*mpy*$_{Mpy}$:*mpy*$_{Mab}$	MabΔ*mpy*$_{Mpy}$ harboring p*mpy*$_{Mab}$	This Study
MabΔ*mpy*$_{Mpy}$:*mpy*$_{Mab}$*	MabΔ*mpy*$_{Mpy}$ harboring p*mpy*$_{Mab}$*	This Study
MabΔ*mpy*$_{Mpy}$:*mpy*$_{Mab}$**	MabΔ*mpy*$_{Mpy}$ harboring p*mpy*$_{Mab}$**	This Study
pMpy$_{Msm}^{**}$	Msm_Mpy with I64A, R93A, V97A, and R128A)	This Study

mc²155	High-Frequency Transformation strain of *M. smegmatis* as parent wild-type	(13)
MsmΔ*mpy*$_{Msm}$	ΔMsmeg_1878 in mc2 155, zeo^{r}	(13)
MsmΔ*mpy*$_{Msm}$::Mpy$_{Msm}$	MsmΔ*mpy*$_{Msm}$ harboring pMpy$_{Msm}$	This Study
MsmΔ*mpy*$_{Msm}$::Mpy$_{Msm}$**	MsmΔ*mpy*$_{Msm}$ harboring pMpy$_{Msm}$**	This Study

Sequence of codon optimized synthetic *mpy*$_{Msm}$:

gttgccacatatgtcaagccattcgatggattcaagccgcaccatggtcgtcgaagacgaccgcgagagcactgctggcac
caccccggaacggccccatgccgaggtcgtcgtcaagggtcgcaacgtcgaggtccccgaccatttccgcacctacgtct
cagagaagctgtcgcgccttgagcgattcgacaagaccgcctacctcttcgacgtcgaacttgaccacgaacgcaaccgc
cgccaacgcaagaactgtcaacacgtcgagatcaccgcgcgcggggccggccctgtcgcccggggcgaggcctgcgccg
acagcttctacaccgctttcgagtcggcggtccaaaagctcgagggcagactgcgacgcgccaaggaccgggccaagatc
cactacggcgacaagacccccgtgtcgctggccgaggccaccgcgaaagacccgctggcgggcctcgacgtgtccaggg
aggacggtgaggcgcgctacaacgacggcgtcgccgagcacgaacccggccggatcgtccgcatcaaggaccaccccg
cgacgcccatgacggtcgacgacgcgctctacgagatggaactggtcggtcacgacttcttcctgttccacgacaaggaga
cggacagaccatcggtggtgtaccgccgtcacgcgttcgactacgggctgatccgcctggccaagcttgccgacc

Sequence of codon optimized synthetic p*mpy*$_{Mab}$*:

ggtacccacgaatcggtggcaaggcgcgtgaacacggactaccgtcggggccaacaagctatgaacgttccgcgtttgggc
ggtcaagccactccccagacacggagcgcctgcttcccgtatggcggaggaggtggttgccaaccccagcgccggcgggc
tgccgtctcagcgtttcaggcgactccggcgtacttcaaatatcaaacatgcgcgccgccaatatctttagtccctagcgggc
aggttggacgcaagcgcactctcagtaccggaggtatgagttgtcaagcacatcaaaggcatccgttgcacttgaggacggg
gagcgggcggcccaggccgatatcgtggtcaagggccgaaacgtagaaatccccgatcacttccgaatctttgtgaacgac
aaactgacccgtctggaacgttacgacaagaccctttacctctttgatgtagaactcgaacacgaacgcaatcgtcgccaga
agaagagctgccagcgcgtggagatcaccgcgcggggccgggggccggttgtccgcggcgaggcatgcgccgccacgttc
gccgccgcgctcgccgctgccgtcgccaagctggaaagtcggctgcgtcggggccgtgaccgccgcaaggttcactacgg
ggacaagacgccggtttcgctcgccgaggccaccgccgtcctgccggccgatgctttcacgccgcccgtcgccgtgaacgg
cgcgcacagtcataacggtgccgaggttgatgatggccacggccccggtcagatcgtgcgcaccaaggaacacccggcca
agccgatgacggtcgatgacgcgctgtacgagatggaactggtcggtcacgactttttcctcttccacgaccagtcgtccggt
cgcccatcggtggtgtaccgcaggcacgcgtatgactacggcctgatacgcctcggggaatagtctaga

Sequence of codon optimized synthetic p*mpy*$_{Mab}$**:

ggtaccccggccgcatcgcgctgcgccggacggcccggccggccacggaattgatgcttgtcgacgacgtggtgaccacc
ggagccacggttgccgaatcggtgcgggtgttgaaatccgcaggatttcgagtcgcggccgcgctcaccctgacccattcct
agacgcatgttggagattgttcgcctgatcggctcatcgcgagtatgacgatctcgaaacgactctcacgaatcggtggcaag
gcgcgtgaacacggactaccgtcggggccaacaagctatgaacgttccgcgtttgggcggtcaagccactccccagacac
ggagcgcctgcttcccgtatggcggaggaggtggttgccaaccccagcgccggcgggctgccgtctcagcgtttcaggcgac
tccggcgtacttcaaatatcaaacatgcgcgccgccaatatctttagtccctagcgggcaggttggacgcaagcgcactctc
agtaccggaggtatgagttgtcaagcacatcaaaggcatccgttgcacttgaggacggggagcgggcggcccaggccgata
tcgtggtcaagggccgaaacgtagaaatccccgatcacttccgaatctttgtgaacgacaaactgacccgtctggaacgtta
cgacaagaccgcctacctctttgatgtagaactcgaacacgaacgcaatcgtcgccagaagaagagctgccagcgcgtgg
agatcaccgcgcggggcgccgggccggttgcccgcggcgaggcatgcgccgacacgttccacagcgcgctcgaagctgc
cgtcgccaagctggaaagtcggctgcgtcggggccgtgaccgcgccaaggttcactacggggacaagacgccggtttcgct
cgccgaggccaccgccgtcctgccggccgatgctttcacgccgcccgtcgccgtgaacggcgcgcacagtcataacggtg
ccgaggttgatgatggccacggccccggtcagatcgtgcgcaccaaggaacacccggccaagccgatgacggtcgatgac
gcgctgtacgagatggaactggtcggtcacgactttttcctcttccacgaccagtcgtccggtcgcccatcggtggtgtaccgc
aggcacgcgtatgactacggcctgatacgcctcggggaatagtctaga

Table S3: List of Oligonucleotides used in this study.

Oligo_ Name	Sequence	Used in:
Mab_Del_Mpy_Left_Fwd2	TACTTGAATATTggtgttgaaatccgc	Δmpy_{Mab}
Mab_Del_Mpy_Left_Rev2	AGTTCTAGAggatgcctttgatgt	Δmpy_{Mab}
Mab_Del_Mpy_Right_Fwd2	CCTAAGCTTggcctgatacgcctc	Δmpy_{Mab}
Mab_Del_Mpy_Right_Rev2	GTAACTAGTgctggctgaccgacg	Δmpy_{Mab}
Mab_Mpy_Fwd	TCTAGAatgttgccggccgcatcg	$\Delta mpy_{Mab}::mpy_{Mab}$
Mab_Mpy_Rev	GTCGACCACCAATCTAGAgggattctctgcgcggtg	$\Delta mpy_{Mab}::mpy_{Mab}$
Mab_Mpy_KpnI_Fwd	GGTACCcacgaatcggtggca	$\Delta mpy_{Mab}::mpy_{Mab}$*, $\Delta mpy_{Mab}::mpy_{Mab}$**
pRT24_Rev2	TCTAGACTATTCCCCGAGGCGTAT	$\Delta mpy_{Mab}::mpy_{Mab}$*, $\Delta mpy_{Mab}::mpy_{Mab}$**
Mab_3583c_Mpy_Fwd10	CAATCGTCGCCAGAAGAAG	RTqPCR
Mab_35b3c_Mpy_Rev10	CGACTTTCCAGCTTGGC	RTqPCR
Mab_3773c_S13_Fwd	GAAATCGCGCTCACCTATATCT	RTqPCR
Mab_3773c_S13_Rev	TGAGAGTGGCTTCGATGTATTC	RTqPCR
Mab_0332c_S14C-_Fwd2	CACAGTCCGAGCTGAACC	RTqPCR
Mab_0332c_S14C-_Rev2	AACACGTACACGGGACAAA	RTqPCR
Mab_1441_L31C+_Fwd	ATGAAATCGGGTATTCACC	RTqPCR
Mab_1441_L31C+_Rev	TTGCGCTTGCCGTAG	RTqPCR
Mab_3009_SigA_Fwd4	CCAAGAACCATCTGCTGGAA	RTqPCR
Mab_3009_SigA_Rev4	CCAGGTTGCCTTCCTGAAT	RTqPCR
